# Unique Aerofoil‐Structured Microfluidics for High Throughput Lipid Nanoparticle Formulation Screening and Scale‐up

**DOI:** 10.1002/advs.202511222

**Published:** 2025-10-28

**Authors:** Dongsheng Liu, Mingzhi Yu, Yuguo Zhang, Allen Mathew, Tianyu Guan, Liang Yao, Xianqing Wang, Wenxin Wang, Nan Zhang

**Affiliations:** ^1^ Centre of Micro/Nano Manufacturing Technology (MNMT‐Dublin) School of Mechanical & Materials Engineering University College Dublin Dublin D04 V1W8 Ireland; ^2^ Department of Aerospace and Mechanical Engineering South East Technological University Carlow R93 V960 Ireland; ^3^ The Centre for Research and Enterprise in Engineering (engCORE) South East Technological University Carlow R93 V960 Ireland; ^4^ Charles Institute of Dermatology School of Medicine University College Dublin Dublin D04 V1W8 Ireland

**Keywords:** aerofoil structures, high‐throughput system, lipid nanoparticles, microfluidics, scale‐up system

## Abstract

Herein, a high‐throughput microfluidic platform is presented that addresses the challenges in lipid nanoparticles (LNPs) formulation screening and scale‐up through the design of micromixers with unique aerofoil structures. These structures are optimized to enhance mixing and LNP formation across a wide flow rate range (0.2–50 mL min^−1^), using minimal reagent volumes (as low as 0.1 mL) up to liter‐scale production. A high‐throughput formulation screening instrument named MiNANO‐form and a microfluidic cartridge with eight parallel channels is introduced, which enables simultaneous synthesis of multiple LNP formulations with excellent quality (size 38–150 nm and polydispersity index (PDI), PDI < 0.2) and reproducibility. In addition, for a seamless scale‐up, the platform consists of a custom‐made modular microfluidic unit, named MiNANO‐scale, capable of continuous LNP production at up to 50 mL min^−1^. The platform's performance is validated through mRNA and pDNA transfection, as well as siRNA uptake studies in HEK293, A549, and CFBE cells.

## Graphical abstract

This study presents aerofoiled microfluidic structures optimized for efficient mixing and lipid nanoparticle (LNP) formation across broad flow rates. Based on this design, two dedicated systems are introduced: MiNANO‐form for high‐throughput formulation screening and MiNANO‐scale for continuous large‐scale production, enabling reproducible, high‐quality LNPs validated with mRNA, pDNA, and siRNA delivery.

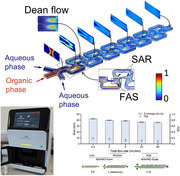

## 1 Introduction

RNA therapeutics represent a revolutionary class of medicines that leverage the unique properties of RNA molecules to prevent and treat diseases.^[^ ^1^, ^2^, ^3^ ^]^ Their rapid integration into mainstream medicine has been notably exemplified by the success of mRNA vaccines developed to combat COVID‐19.^[^ ^4^, ^5^ ^]^ However, challenges such as RNA instability, degradation, immunogenicity, and inefficient delivery remain major hurdles.^[^ ^6^ ^]^ Lipid nanoparticles (LNPs) have emerged as the most effective delivery platform, protecting RNA and enabling targeted delivery.^[^ ^7^ ^]^ Despite progress, only a few LNP‐based RNA therapeutics, e.g., Onpattro, Pfizer/BioNTech's BNT162b2, and Moderna's mRNA‐1273, have received FDA approval, underscoring the complexity of LNP development.^[^ ^8^, ^9^, ^10^ ^]^

Successful LNP‐based therapeutics rely on the screening of a large number of ionizable lipids to establish structureactivity relationships,^[^ ^11^, ^12^, ^13^ ^]^ followed by the systematic optimization of formulations LNPs of selected candidate lipids for satisfying delivery performance.^[^ ^14^, ^15^, ^16^ ^]^ For example, over 300 ionizable lipids were screened to identify the optimal one for Onpattro.^[^ ^8^ ^]^ LNP performance is influenced by lipid composition (ionizable lipids, helper lipids, cholesterol, PEG‐lipids),^[^ ^17^, ^18^, ^19^, ^20^ ^]^ molar ratios,^[^ ^21^ ^]^ lipids and RNA concentrations,^[^ ^22^ ^]^ flow conditions,^[^ ^23^ ^]^ and post‐processing steps,^[^ ^24^, ^25^ ^]^ making optimization a complex and iterative process. In every clinical application of lipid nanoparticle formulations, the lipid composition must be optimized to account for target cell specificity, immune response, and related factors.^[^ ^26^, ^27^, ^28^ ^]^ Therefore, finding the optimal combination is an expensive, tedious, and time‐consuming process. While machine learning (ML) and artificial intelligence (AI) offer potential to accelerate formulation,^[^ ^14^, ^21^, ^29^ ^]^ their success depends on large, high‐quality datasets, necessitating high‐throughput platforms. Traditional pipette mixing lacks precise control and yields poor batch consistency,^[^ ^30^ ^]^ while automated systems offer higher throughput but often produce larger, heterogeneous nanoparticles and face scalability issues.^[^ ^31^ ^]^ These limitations underscore the need for reliable high‐throughput and scalable manufacturing technologies to support data‐driven formulation and clinical translation.

Microfluidics has emerged as a promising solution, providing precise control over nanoparticle characteristics—size, polydispersity index (PDI), and encapsulation efficiency (EE)—with potential for scale‐up.^[^ ^32^, ^33^ ^]^ Central to this is the microfluidic mixer, which rapidly combines organic and aqueous phases to drive uniform LNP self‐assembly. Efficient mixing ensures homogeneous nucleation and controlled nanoparticle growth, which are key to forming stable, monodisperse nanoparticles and minimizing RNA waste.^[^ ^34^ ^]^ Several mixer designs have been developed, each with strengths and limitations. T‐mixer^[^ ^35^ ^]^ creates turbulent mixing but suits large volumes better. Hydrodynamic focusing flow (HFF)^[^ ^36^ ^]^ allows precise diffusion‐based mixing, though with scalability issues. Staggered herringbone mixers (SHM)^[^ ^37^ ^]^ enhance mixing via microgrooves but are clog‐prone. The iLiNP system^[^ ^38^ ^]^ uses baffles to generate vortices but has high‐pressure requirements and clogging risks. More advanced designs, such as the Tesla mixer^[^ ^39^ ^]^ and Dean vortex bifurcating mixer (DVBM)^[^ ^40^ ^]^ exploit split‐and‐recombination (SAR) of flow for efficient mixing but usually require high flow rates (>10 mL min^−1^), resulting in waste and poor performance at lower scales for formulation screening. Hybrid systems exist but often suffer from clogging, poor flow flexibility, or scale‐up difficulties.

To support RNA LNP formulation needs, various commercial microfluidic platforms (e.g., NanoAssemblr Spark, Ignite, Blaze,^[^ ^41^ ^]^ NanoGenerator Flex‐S,^[^ ^42^ ^]^ Sunscreen, and Sunbather^[^ ^43^ ^]^) have been introduced. Yet, most are low in throughput, single‐channel systems with limited capacity for high‐throughput synthesis and face challenges in avoiding cross‐contamination. Critically, they lack the ability to seamlessly transition from screening to large‐scale production, which is essential for AI‐driven formulation and rapid clinical translation.

To address these challenges, we have developed a high‐throughput microfluidic platform that overcomes the current limitations in RNA‐based therapeutics by enabling efficient LNP screening, optimization, and scale‐up. The backbone of this platform is a novel microfluidic mixing design featuring unique aerofoil structures, facilitating enhanced mixing and precise nanoparticle control across a wide range of flow rates. We designed a high‐throughput microfluidic cartridge with 8 parallel channels, with each channel having aerofoil mixing structures, enabling simultaneous synthesis of eight LNP batches, streamlining screening and optimization processes. The cartridge is fully compatible with standard 96‐well plates and 8‐channel pipettes, facilitating seamless integration into laboratory workflows. To support this platform, we developed the MiNANO‐form, a dedicated LNP synthesis instrument for the eight‐channel high‐throughput cartridge. Additionally, for large‐scale production, we created the MiNANO‐scale, a scalable system with a compatible cartridge capable of operating at flow rates up to 50 mL min^−1^ while maintaining consistent LNP size and PDI. This scale‐up cartridge has the same aerofoil structures but enlarged for higher flow rates. Together, these advancements enable efficient, scalable, and reproducible LNP synthesis, addressing the critical gap between small‐scale screening and large‐scale production in RNA‐based therapeutic development.

**Figure 1:**
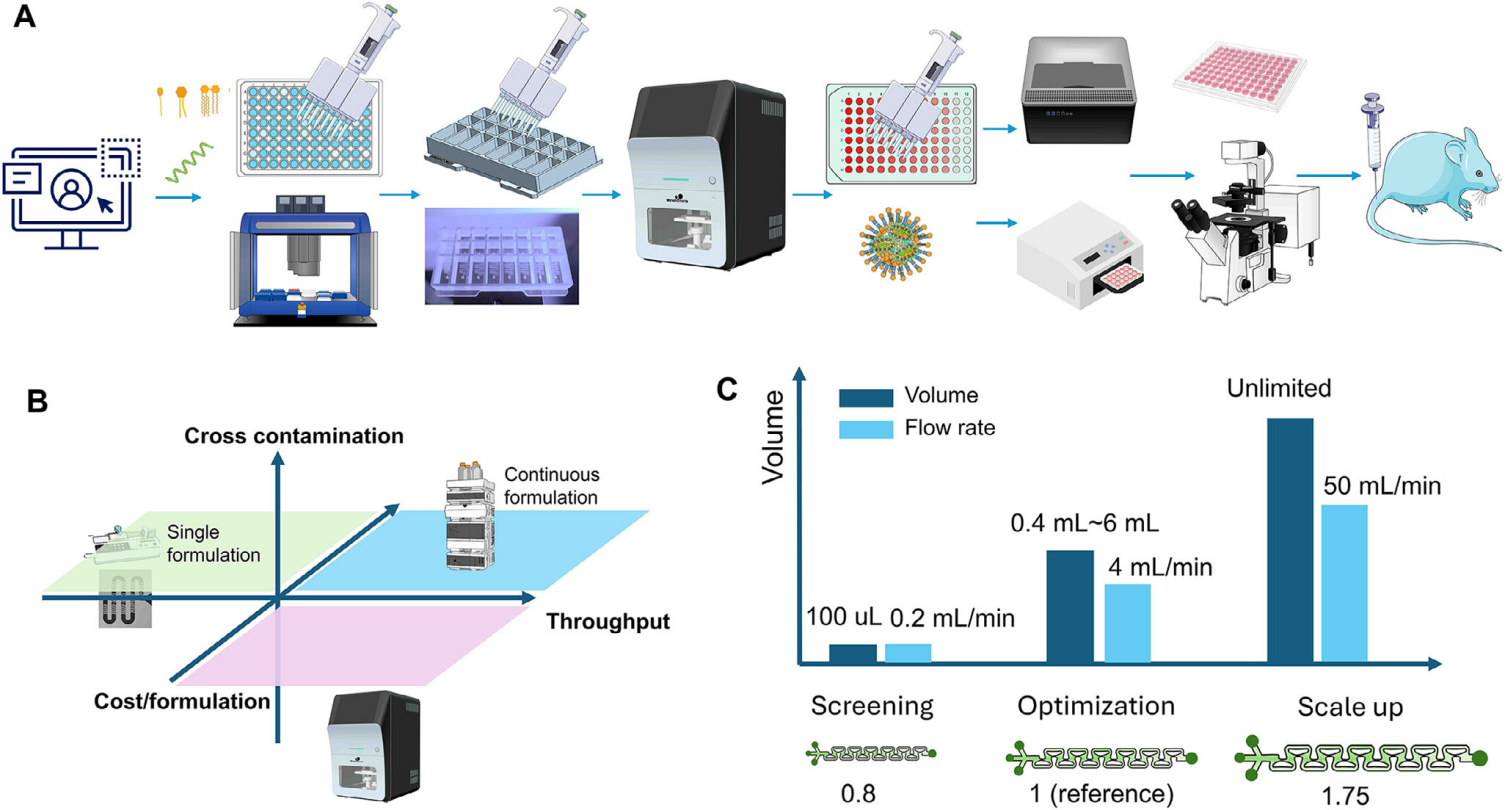
Overview and capabilities of the high‐throughput LNP platform. A) An overview of the LNP workflow (LNP screening to animal testing) using our high throughput platform; B) Positioning the high throughput platform in terms of throughput, cost, and cross‐contamination with respect to existing technologies; C) Adaptability of aerofoil structures for different flow rates and volume.

In this article, we investigated mixing mechanisms at low, medium, and high flow regimes (0.2–50 mL min^−1^) to support both formulation discovery with small volume using MiNANO‐form (as low as 0.1 mL) and scale‐up for large volume production using MiNANO‐scale. An overview of the LNP workflow (LNP screening to animal testing) using our high‐throughput platform is shown in **Figure** **1**A. The platform is designed to accelerate the workflow with zero cross‐contamination and reduce the cost per formulation (Figure 1B). The aerofoil structures can be scaled down to synthesize a small volume (minimum 0.1 mL) of LNPs at low flow rates and scaled up to synthesize large volumes (liters) of LNPs at high flow rates, maintaining similar size and PDI (Figure 1C). The quality of LNPs produced by our platform was validated through cellular assays, including GFP mRNA and GFP pDNA transfection as well as siRNA uptake studies in HEK293, A549, and CFBE cell lines. These results confirm that our system enables high‐throughput formulation screening, scalable RNA‐LNP manufacturing, providing a critical infrastructure for AI‐driven formulation discovery and accelerating the translation of RNA nanomedicines from bench to clinic.

## 2 Results

### 2.1 Design and Optimization of Aerofoil Structure Microfluidic Mixer

**Figure 2:**
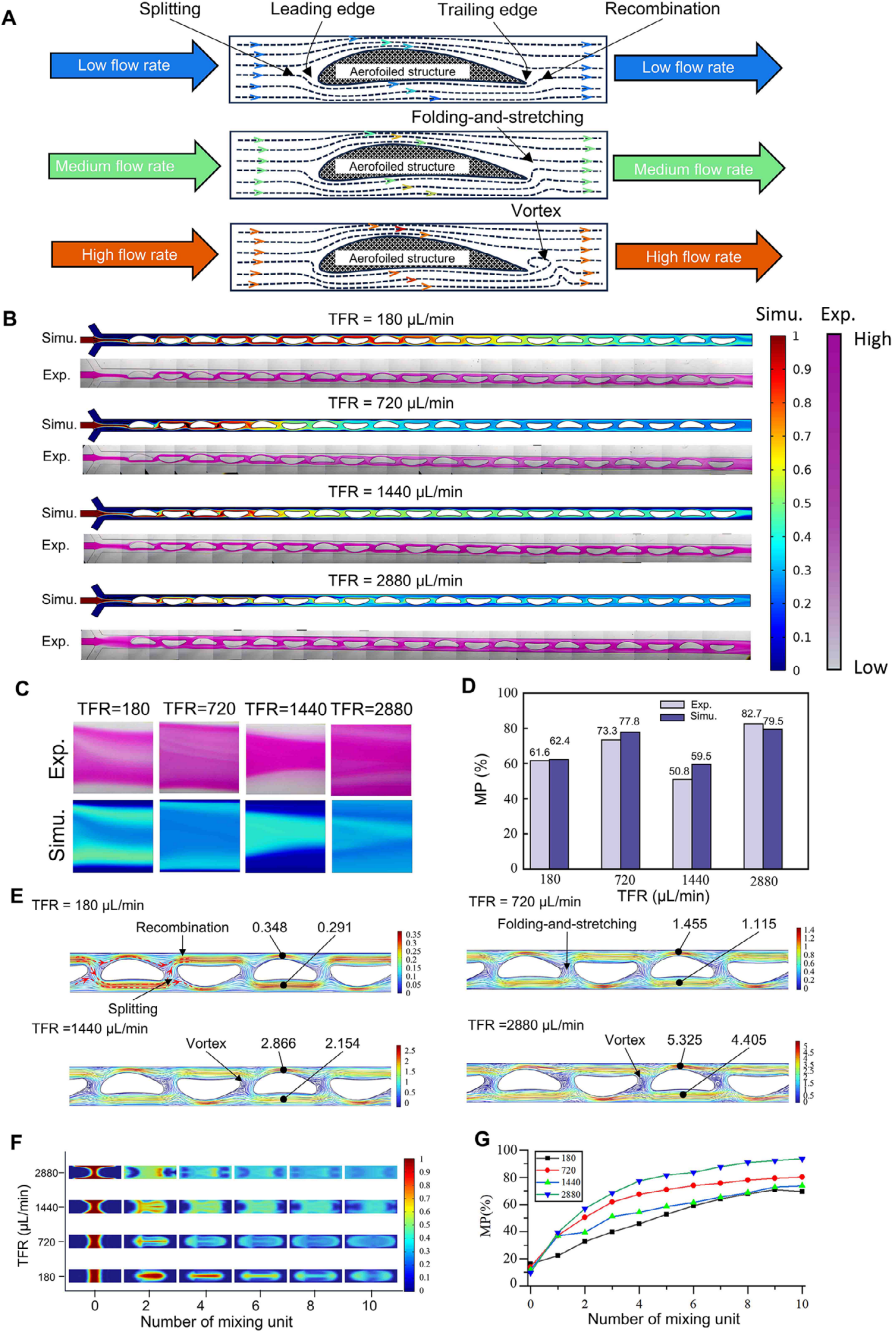
Design of an aerofoil structure microfluidic cartridge and validation of the simulation model through mixing experiments. A) Schematic of the mixing mechanism of the cartridge as the flow rate increases; B) Experimental and simulation results. In the simulations, blue and red correspond to normalized concentrations of 0 and 1, respectively. In the experiments, transparent and pink regions represent low and high Rhodamine‐dyed concentrations, respectively; C) Longitudinal section of concentration at outlet; D) Mixing performance (MP) calculation and image processing for simulation results; E) Velocity streamline plots; F) Cross section of concentration at outlet, showing how mixing progresses as the flow passes through the mixing structures; G) Mixing performance (MP) of cross section at outlet, quantitatively illustrating how mixing progresses through the structures. Total flow rate (TFR) is indicated in the plots.

Inspired by the capability of the aerofoil structure of aircraft wings to generate a pressure differential, we have designed microchannels integrated with aerofoil structures, dividing the channels into two bypass pathways, as illustrated in **Figure** **2**A. This unique design enables highly efficient mixing across low, medium, and high flow rates. At low flow rates, mixing is achieved by the SAR mechanism, where the flow splits at the leading edge and recombines at the trailing edge. SAR enhances mixing by increasing flow laminates and expanding the contact surface of fluid layers, while performing baker's transformations to evenly redistribute concentrations.^[^ ^44^ ^]^ When the flow rate increases, a folding‐and‐stretching (FAS) of flow emerges around the trailing edge due to the pressure difference between the upper and lower surfaces of the aerofoil structure. In FAS, fluid layers are folded, bringing regions with high and low concentrations into close proximity, while stretching elongates fluid layers, thinning them and increasing their surface area.^[^ ^45^ ^]^ Further increase of flow rates can induce the localized vortices due to pressure gradients under laminar flow conditions. These vortices further enlarge the contact area between fluid layers and amplify FAS, significantly enhancing mixing efficiency.^[^ ^46^ ^]^

The mixing mechanisms were validated through CFD simulations and experiments (Figure S1, Supporting Information). The experimental and simulation results are presented as Figure 2B. Mixing performance values were calculated using the image‐based method described in the Methods section. Outlet images were analyzed both qualitatively and quantitatively, demonstrating strong agreement between experimental and simulated results across all tested flow rates (Figure 2C,D). The relative errors between simulation and experiment are 1.29%, 6.13%, 17.12%, and 3.86% at total flow rates of 180, 720, 1440, and 2880 µL min^−1^, respectively. All absolute errors remain below 10%, with the maximum error of 8.7% observed at 1440 µL min^−1^. At 180 µL min^−1^, aerofoil structures induce SAR flows, and their alternating orientation promotes fluid exchange between upper and lower bypass channels (indicated by the red dashed arrow in Figure 2E), enhancing mass transfer and mixing efficiency. At this total flow rate, the flow velocity over the upper (convex) surface is 19.6% higher than the lower (concave) surface. As the total flow rate increases to 720 and 1440 µL min^−1^, the velocity difference rises to 30.4% and 33.2%, respectively, intensifying FAS and initiating vortex formation near the trailing edge. At 2880 µL min^−1^, vortices become more pronounced, further promoting mixing through enhanced interfacial contact.

A distinct interface between fluids with concentrations of 1 and 0 is observed across the cross‐section prior to encountering the aerofoil structures (i.e., at 0 mixing units, Figure 2F), resulting from diffusion driven by the initial concentration gradient. As the total flow rate increases, the sheath flows with a concentration of 0 more strongly squeeze the central stream with a concentration of 1,^[^ ^47^ ^]^ producing increasingly curved and hyperbolic concentration contours, as shown in Figure 2F at 0 mixing units. As the number of mixing units increases, the concentration distribution becomes progressively more uniform across the cross‐section, as indicated by colour gradients, leading to a steady increase in the mixing performance value (Figure 2G). Notably, at 1440 µL min^−1^, the mixing performance is lower than that at 720 µL min^−1^. This decrease is primarily due to the wall jet effect,^[^ ^48^ ^]^ where the high‐concentration fluid flows around the surface of the aerofoil structures without significant mass exchange with the adjacent low‐concentration bypass flows (Figure 2B). This behavior is also evident from the cross‐sectional plots in Figure 2F, which show limited mixing in the central region. Based on these understandings, we have further optimized aerofoil structures and channel configurations.

**Figure 3:**
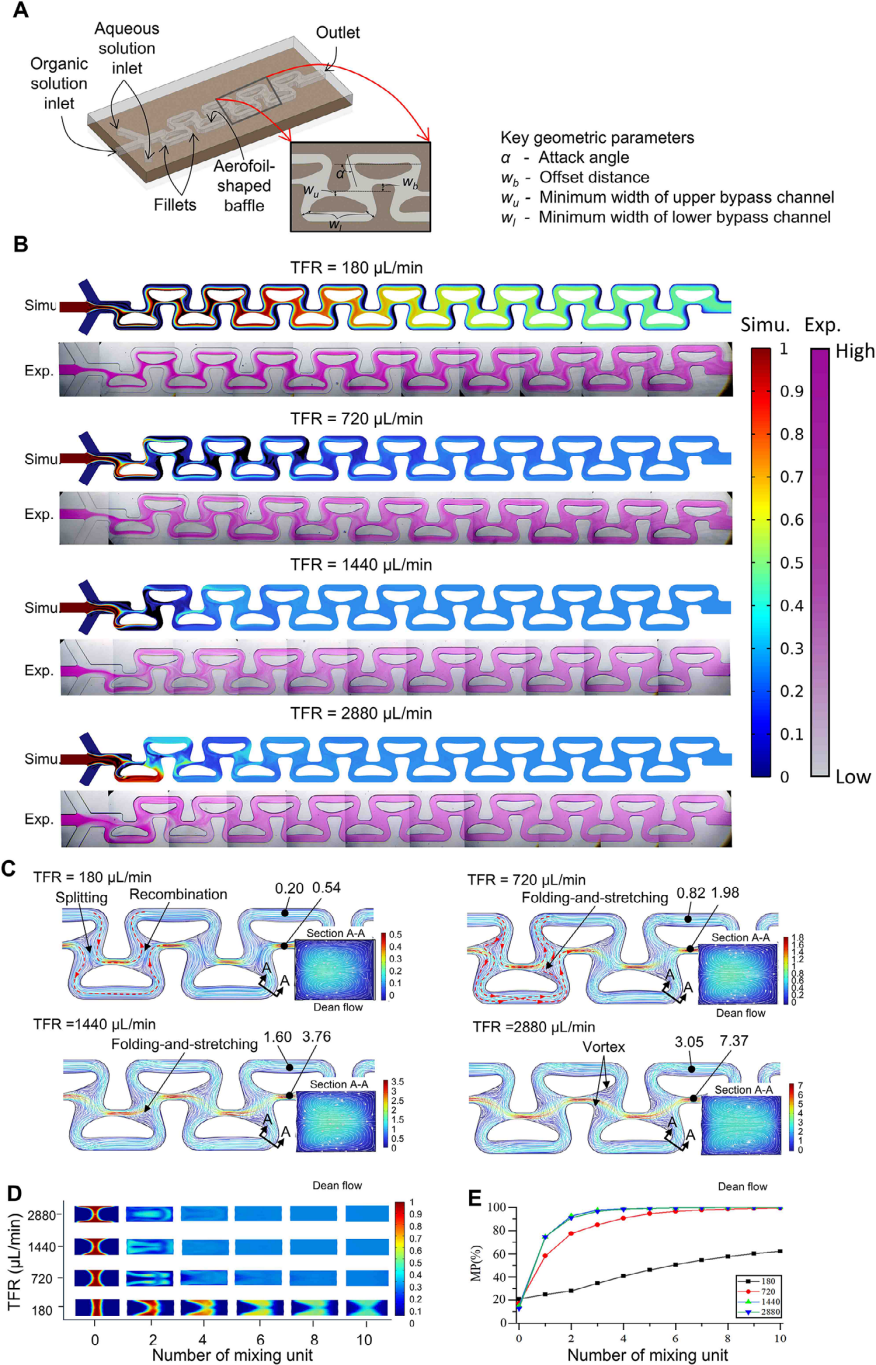
Experimental and simulation results of an optimized aerofoil structure microfluidic channel. A) Geometric design of the microfluidic cartridge; B) Experimental and simulation results. In the simulations, blue and red correspond to normalized concentrations of 0 and 1, respectively. In the experiments, transparent and pink regions represent low and high Rhodamine‐dyed concentrations, respectively; C) Velocity streamline plots for the cartridge configuration 105/60/120/100; D) Cross section of concentration at outlet; E) Mixing performance (MP) at the outlet for the cartridge configuration 105/60/120/100, where mixing stabilizes rapidly, reaching complete mixing (normalized Mixing Performance≈ 1). Total flow rate (TFR) is indicated in the plots.

The inefficiency in mixing induced by the wall jet effect was resolved by modifying the aerofoil structures as shown in **Figure** **3**A. HFF was employed to enhance mixing efficiency by creating two fluid interfaces. The attack angle α in ° is defined as the angle between the flow direction and the chord line. Each aerofoil structure divides the microchannel into two bypass channels: the upper bypass channel features a contraction–expansion profile with a minimum width denoted as *w_u_* (µm), like a Venturi tube, while the lower bypass channel has a relatively uniform width with a minimum width *w_l_* (µm) near both the leading and trailing edges. The offset distance between the top sides of the upper bypass channels of two adjacent baffles, measured along the aerofoil structure's thickness direction, is denoted as *w_b_* (µm). The channel design is characterized by the geometrical parameters as “*α*/*w_u_*/*w_l_*/*w_b_*”. The geometric parameters, particularly the attack angle (*α*) and the width ratio of the lower to upper bypass channels (*w_l_*/*w_u_*), play a crucial role in optimizing mixing performance.

To address the wall jet effect at the solid boundary of the aerofoil structure, curved channels with fillets are incorporated into the outer walls of the lower bypass channel around the leading and trailing edges of the aerofoil structure, as shown in Figure 3A. When the geometrical parameters were 105/60/120/100, mixing results aligned well with simulations (Figure 3B). The flow split point shifts further rearward (Figure 3C), intensifying the FAS effect, evident from increased streamline distortion near trailing edges. The cross‐section and mixing performance value are shown in Figure 3D,E. At 180 µL min^−1^, as the lower bypass stream flow no longer splits and flows directly into the upper bypass channel, the mixing performance deteriorates despite stronger FAS. As the total flow rate increases to 720 µL min^−1^, the larger velocity difference between the upper and lower bypass channels further strengthens the FAS effect, leading to improved mixing performance. At 1440 µL min^−1^, vortex formation further boosts mixing.

In addition to steady‐state simulations, transient CFD analyses were also performed to capture flow evolution around the aerofoil structures at different total flow rates (180, 720, 1440, and 2880 µL min^−1^). These simulations reveal that the system reaches a stable flow regime rapidly: for example, the transition period at 180 µL min^−1^ is ≈0.088 s, and decreases substantially at higher flow rates. Thus, the transient phase accounts for only a very small fraction of the total run time and has minimal impact on overall mixing performance. Representative snapshots of vortex development and interface deformation during the transient phase are provided in Figure S2 (Supporting Information), further supporting the mechanistic interpretations of FAS and vortex‐enhanced mixing.

Compared to a DVBM mixer with the same main channel size and a comparable overall length, which achieves full mixing at 2880 µL min^−1^ via SAR and Dean flow (Figure S3, Supporting Information), our design achieves complete mixing at just 720 µL min^−1^, demonstrating superior mixing performance at a low flow rate.

### 2.2 High Throughput 8‐Channel Microfluidic Cartridge and System Development for LNP Synthesis

**Figure 4:**
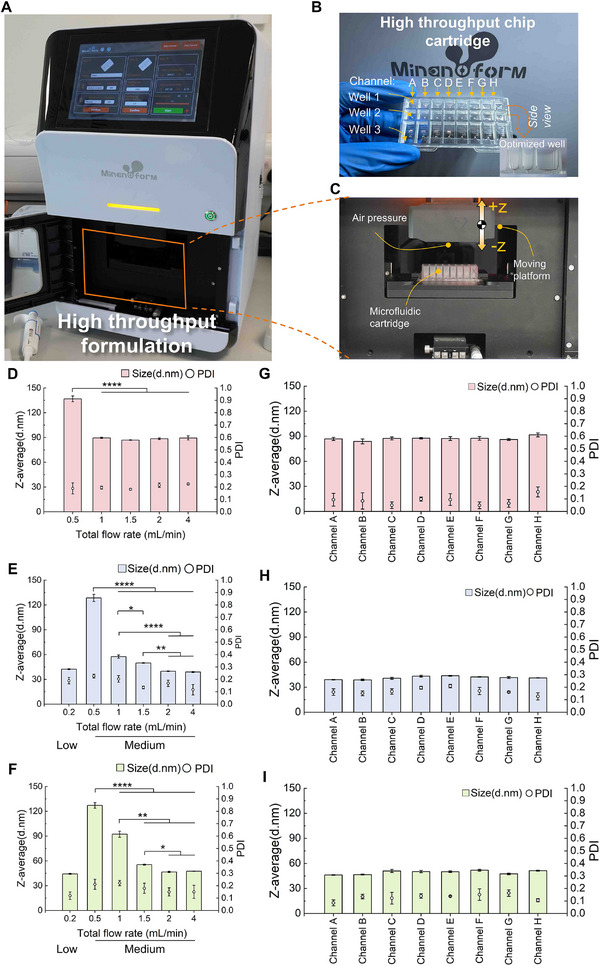
High‐throughput LNP formulation system (MiNANO‐form). A) Photograph of the MiNANO‐form formulation instrument; B) High throughput cartridge featuring eight independent microfluidic channels, each connected to individual inlets (Wells 1–3); C) Integration of the cartridge into the platform, highlighting the pressure inputs and sealing mechanism; D–F) Effects of varying total flow rate on LNP size and PDI using different ionizable lipids: (D) DDAB, (E) SM‐102, and (F) ALC‐0315; G–I) Size and PDI of LNPs produced from all eight channels at a total flow rate of 2 mL/min with different ionizable lipids: (G) DDAB, (H) SM‐102, and (I) ALC‐0315.

To enable high‐throughput screening and optimization of LNP formulations, a cyclic olefin copolymer (COC) plastic microfluidic cartridge consisting of 8 parallel mixing channels was designed to enable the simultaneous synthesis of 8 distinct formulations independently. The size and alignment of the 8‐channel cartridge are compatible with industrial 96‐well plates and 8‐channel pipettes (**Figure** **4**A–C). The prepared aqueous and organic phases can be easily transferred into the input wells in the cartridge (wells 1 and 2 in Figure 4B) and fully mixed in the microchannels incorporated with the aerofoil structures for nanoparticle synthesis.

As part of the high‐throughput microfluidic platform, a standalone instrument named MiNANO‐form has been developed to facilitate high‐throughput screening of LNP formulation. MiNANO‐form is designed for the 8‐channel microfluidic cartridge, which drives the aqueous and organic phases for rapid and uniform mixing through the aerofoil structured mixer. The synthesized LNP solution is collected in the output well in the cartridge (well 3 in Figure 4B). The design combination of MiNANO‐form and 8‐channel cartridge is refined in such a way that LNP synthesis can be done using small volumes of aqueous and organic phase (a few hundred microliters) at very low flow rates up to 0.2 mL min^−1^. For formulation screening, only small volumes of organic and aqueous phases, typically a few hundred microliters, are needed for physicochemical and in vitro characterization. Consequently, low flow rates are preferred, as high flow rates require fast control system responses and longer times to reach steady‐state flow conditions that are impractical for small‐volume experiments of a few hundred of µL. Our high‐throughput platform allows flexible adjustment of lipid/payload composition and molar ratios, enabling faster, cost‐effective formulation discovery.

The advantage of an aerofoil structure is that it can be scaled down for small volume synthesis at low flow rates and scaled up for large quantity synthesis at higher flow rates without compromising the quality of LNPs. Two types of 8‐channel cartridges with aerofoil structures were designed to cover the low (0.2–0.5 ml min^−1^) and medium flow rate (0.5–4 ml min^−1^) range. For the low flow rate cartridges, the size of the aerofoil structures is scaled down 0.8‐fold as shown in Figure 1C.

To assess the effect of total flow rate on LNP characteristics, total flow rates from 0.5 to 4 mL min^−1^ were tested (see results in Figure 4D–F) using the standard cartridge (Figure 1C middle). DDAB‐LNPs showed a size reduction from 136.83 to 89.54 nm, plateauing thereafter, suggesting a stable size was reached. SM‐102‐LNPs and ALC‐0315‐LNPs exhibited sharper declines, reaching ≈40– ≈47 nm, respectively, with PDIs dropping below 0.2 beyond 2 mL min^−1^. As further increases in total flow rate did not significantly reduce particle size, 2 mL/min was selected for reproducibility assessment. At this total flow rate, LNPs synthesized across eight channels using DDAB, SM‐102, and ALC‐0315 lipids showed minimal variation (shown in Figure 4G–I; Table S1, Supporting Information). DDAB‐LNPs ranged from 83.84–91.68 nm (CV 2.48%) with an average PDI of 0.086. SM‐102‐ and ALC‐0315‐LNPs had mean sizes of 41.28 nm (CV 4.37%) and 49.32 nm (CV 4.52%), with PDIs of 0.167 and 0.130, respectively. These results demonstrate that our high‐throughput formulation system enables effective size control and high inter‐channel reproducibility for various LNP formulations. In addition, in order to demonstrate the benefits of our design for low‐volume and low total flow rate screening, we further reduce the total flow rate down to 0.2 mL/min with the scale‐down cartridge for both SM‐102 and ALC‐0315 (Figure 4E,F). The size of LNPs under both formulations showed a good comparability with the standard cartridge when total flow rates reached 2 mL/min, indicating a successful formulation screening using a scale‐down chip. This enables our MiNANO‐form platform for extremely small volume formulation down to 100 µL to save on expensive genetic materials.

**Figure 5:**
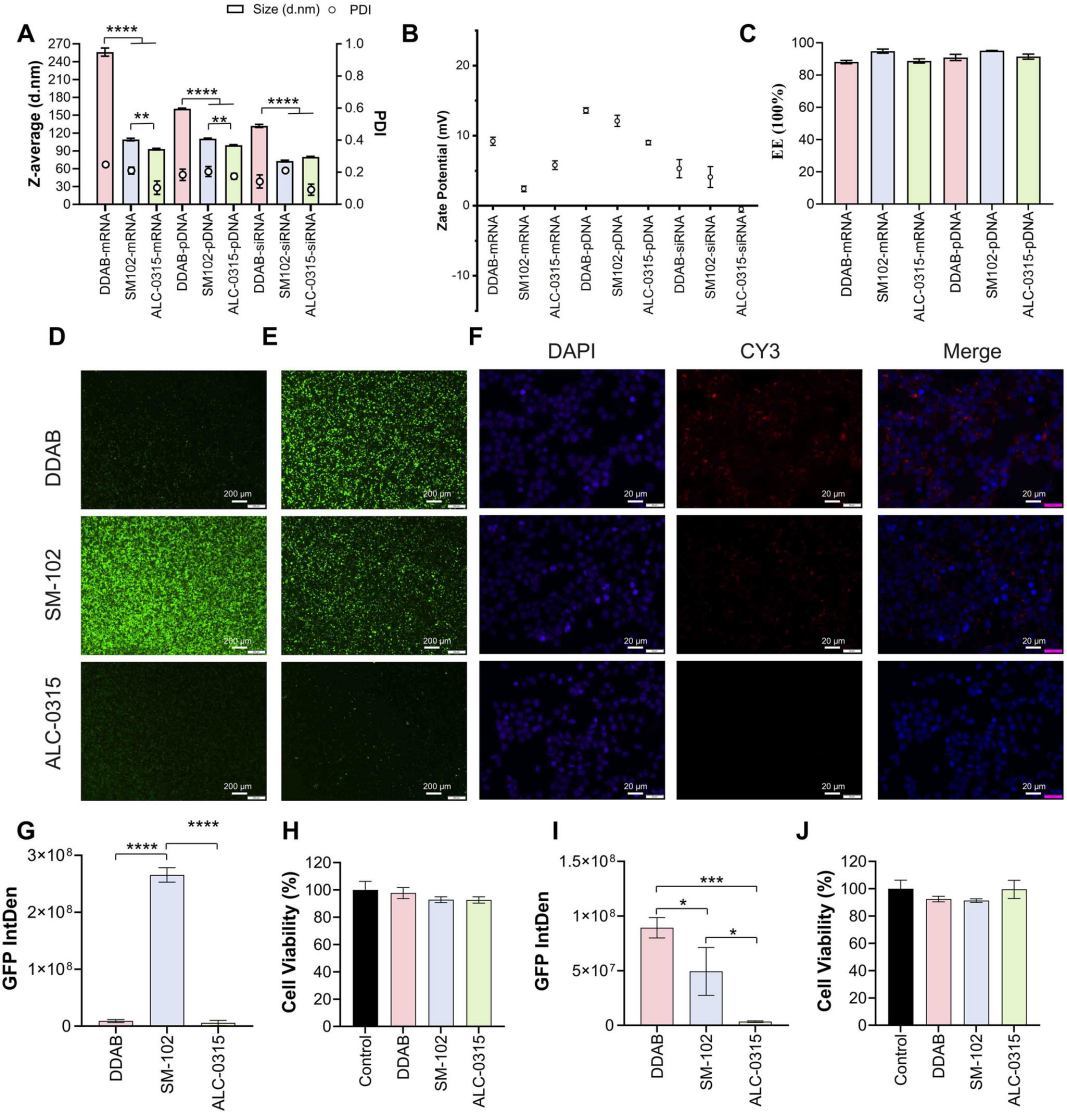
In‐vitro test performance using different LNPs by MiNANO‐form. A) Size and PDI results of GFP mRNA‐LNPs, GFP pDNA‐LNPs, and siRNA‐LNPs; B) Zeta‐potential results of GFP mRNA‐LNPs, GFP pDNA‐LNPs, and siRNA‐LNPs; C) Encapsulation efficiency results of GFP mRNA‐LNPs and GFP pDNA‐LNPs; D) Representative fluorescence microscopy images of GFP expression in HEK cells transfected with GFP mRNA‐LNPs; E) Representative fluorescence microscopy images of GFP expression in HEK cells transfected with GFP pDNA‐LNPs; F) Fluorescence microscopy images showing the uptake of siRNA‐LNPs in HEK cells; G) Semi‐quantitative analysis of integrated fluorescence intensity related to GFP expression in HEK cells transfected with GFP pDNA‐LNPs; H) Cell viability results in HEK cells transfected with GFP mRNA‐LNPs; I) Semi‐quantitative analysis of integrated fluorescence intensity related to GFP expression in HEK cells transfected with GFP pDNA‐LNPs; J) Cell viability results of HEK cells transfected with GFP pDNA‐LNPs.

To assess the system's performance of payload encapsulation and in vitro performance, we evaluated three LNP formulations (DDAB, SM‐102, ALC‐0315) loaded with mRNA, pDNA, or siRNA. Among mRNA‐LNPs, ALC‐0315 gave the smallest size (92.96 nm) and lowest PDI (0.103), while DDAB showed the largest size (256.1 nm) and highest PDI (0.248) (**Figure** **5**A). Zeta potentials were all below 10 mV (Figure 5B), and mRNA EE exceeded 85% (Figure 5C). For pDNA‐LNPs, DDAB produced the largest particles (160.74 nm), followed by SM‐102 (110.45 nm) and ALC‐0315 (99.56 nm), with slightly higher PDIs (0.175–0.204) and EEs ≈90% (Figure 5A,C). siRNA‐LNPs showed a similar trend, with DDAB‐LNPs being the largest (131.89 nm), followed by SM‐102 and ALC‐0315 formulations (Figure 5A). Transfection efficiency was assessed via GFP expression. SM‐102‐mRNA‐LNPs showed the highest transfection in HEK and A549 cells, while DDAB and SM‐102 formulations were similarly effective in CFBE cells (Figure 5D,G; Figure S4a,d,h,k, Supporting Information). All mRNA‐LNPs maintained high cell viability (>90%) (Figure 5H; Figure S4e,l, Supporting Information). For pDNA delivery, DDAB‐LNPs achieved the highest transfection in HEK and A549 cells, while SM‐102 was superior in CFBE cells (Figure 5E,I; Figure S4b,f,i,m, Supporting Information). Cell viability remained unaffected across groups (Figure 5J; Figure S4g,n, Supporting Information). Cellular uptake was evaluated using Cy3‐labeled siRNA. DDAB‐siRNA‐LNPs showed the strongest fluorescence signal across all cell types, indicating superior uptake, followed by SM‐102 and ALC‐0315 (Figure 5F; Figure S4c,j, Supporting Information). These results demonstrate the commendable performance of our high‐throughput screening system, MiNANO‐form and 8‐channel cartridge with aerofoil structure in synthesizing LNP with consistent quality.

### 2.3 Scale‐Up System Development and Validation

**Figure 6:**
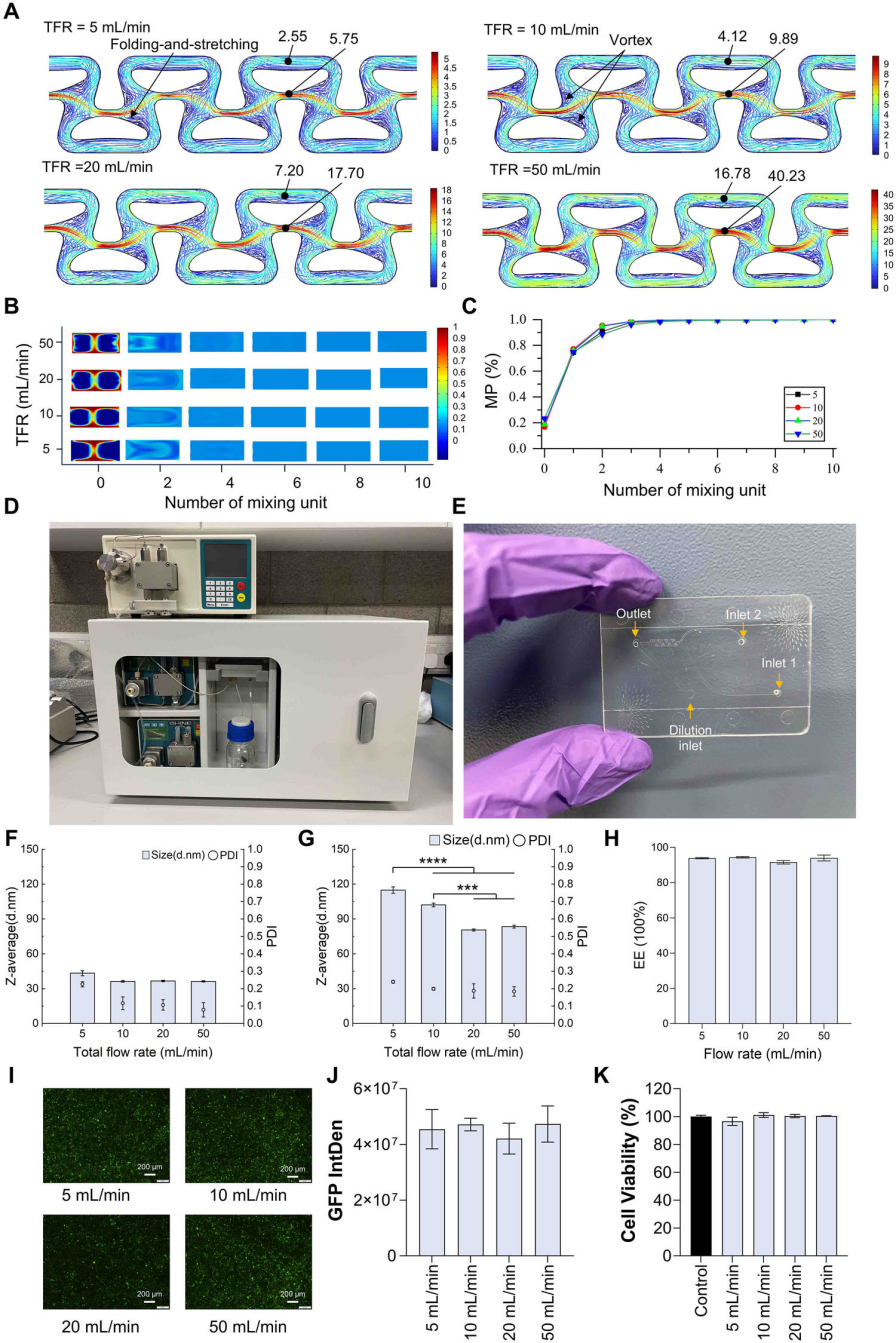
Scalable LNP formulation system (MiNANO‐scale) and its application. A) Velocity streamline plots for the scale‐up cartridge at different flow rates. In the simulation, blue represents a concentration of 0 and red represents a concentration of 1, as indicated by the color bar; B) Cross section of concentration at outlet; C) Mixing performance (MP) at the outlet for scale‐up cartridge; D) Photograph of the MiNANO‐scale formulation platform; E) High throughput cartridge of MiNANO‐scale; F) Size and PDI results of empty SM‐102‐LNPs at different flow rate; G) Size and PDI results of GFP pDNA‐SM‐102‐LNPs at different flow rate; H) Encapsulation efficiency results of GFP pDNA‐SM‐102‐LNPs; I) Representative fluorescence microscopy images of GFP expression in HEK cells transfected with GFP pDNA‐LNPs; J) Integrated fluorescence intensity related to GFP expression semi‐quantified in HEK cells transfected with SM‐102‐pDNA‐LNPs; K) Cell viability results in HEK cells transfected with SM‐102‐pDNA‐LNPs. Total flow rate (TFR) is indicated in the plots.

To facilitate seamless translation from high‐throughput screening to scalable manufacturing, we developed a continuous microfluidic platform, MiNANO‐scale (**Figure** **6**D), and a cartridge (Figure 6E) where the mixing channel architecture was enlarged by 1.75‐fold as shown in Figure 1C while preserving the mixing dynamics of the microchannel used in MiNANO‐form. CFD (Figure 6A,B) showed that concentration profiles reached homogeneity within four mixing units (1 unit = a pair of aerofoil structures) across all tested flow rates, as reflected by mixing performance values approaching 1 (Figure 6C). Accordingly, the scale‐up cartridge was designed with four mixing units. MiNANO‐scale integrates dual peristaltic pumps, enabling flow rates up to 50 mL/min and supporting liter‐scale production. The system ensures continuous, stable mixing while maintaining formulation fidelity under high throughput conditions.

To validate system performance, SM‐102‐based LNPs were synthesized at 5–50 mL min^−1^ (Figure 6F). Particle size decreased from 43.25 nm (PDI 0.225) at 5 mL min^−1^ to ≈36 nm (PDI ≈0.1) at ≥10 mL min^−1^, indicating enhanced mixing efficiency. GFP‐pDNA‐loaded SM‐102 LNPs were also produced (Figure 6G), with particle size decreasing from 114.86 to 80.57 nm between 5 and 20 mL min^−1^, while encapsulation efficiency remained >90% across all flow rates (Figure 6H). Functional assays confirmed consistent transfection efficiency and cell viability, with no significant variation across various flow conditions (Figure 6I–K).^[^ ^31^ ^]^ These results demonstrate that MiNANO‐scale enables robust, scalable synthesis of functional LNPs, directly bridging discovery‐stage optimization with manufacturing‐scale production.

## 3 Discussion

**Figure 7:**
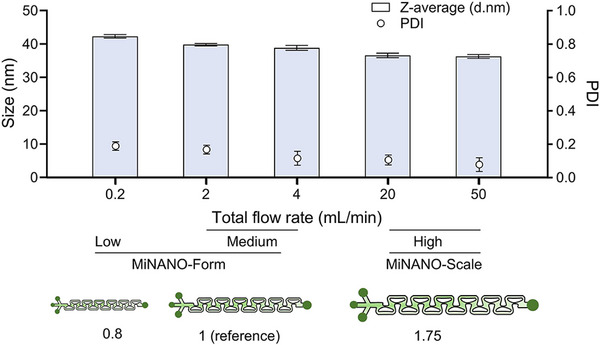
Comparison of the size and PDI of SM102 synthesized at different total flow rates. The average size and PDI were 38.7 nm ± 2.5 and 0.131 ± 0.045 respectively, indicating the adaptability of aerofoil structures for a wide range of flow rates.

We designed and optimized a microfluidic cartridge incorporating aerofoil structures, where the mixing is governed by SAR, FAS, and vortex‐induced mixing mechanisms. These mechanisms are driven by the pressure difference generated around the aerofoil structures. Unlike conventional microfluidic mixers that typically rely on a single mixing mechanism (e.g., DVBM mixers, which work effectively only at high flow rates via Dean flow), our design leverages the aerodynamic profile of the aerofoil structures to actively shape internal flow patterns under laminar conditions. At low flow rates, SAR dominates with the help of local dean flow; with increasing flow rates, FAS becomes more prominent due to pressure difference across the aerofoil structure surfaces; and at even higher flow rates, localized vortex formation further enhances mixing along with Dean flow. This multiscale mixing approach enables efficient, uniform mixing across a broad range of flow rates. To understand the adaptability of aerofoil structures for a wide range of flow rates, the size and PDI of LNPs (SM102) synthesized at different flow rates are compared in **Figure** **7**. Results demonstrate the strong performance of aerofoil structures, enabling consistent LNP synthesis with size: size 38.7 nm ± 2.5 and PDI: 0.131 ± 0.045, across a wide range of flow rates (0.2–50 mL min^−1^) and solution volumes (from small‐scale to large‐scale production). Furthermore, the cartridge's 2D geometry facilitates both scale‐up and scale‐down, and is easy to fabricate, making it well‐suited for the precise formulation and scalable manufacturing of nanoparticles for RNA therapeutics, thus providing a robust bridge between bench‐scale research and scale‐up production.

Our high‐throughput microfluidic system, MiNANO‐form offers a transformative advantage for RNA therapeutic development by addressing the complexities of nanoparticle formulation with enhanced speed, precision, consistency, and scalability. RNA therapeutics demand fine‐tuned delivery systems that balance multiple variables, such as lipid composition, composition ratio, and mixing conditions, that significantly influence efficacy, safety, and stability. Our system enables the rapid screening of hundreds to thousands of formulation conditions, accelerating the identification of optimal combinations for diverse therapeutic targets. This is particularly advantageous in personalized medicine applications, such as custom mRNA vaccines or treatments for rare genetic diseases. Integrated with a high‐throughput DLS platform operating on 96‐well plates, the system allows real‐time characterization of particle size and PDI. Up to 25 distinct LNP formulations can be synthesized and analyzed within 1 hour, and the workflow is shown in Figure S5 (Supporting Information), significantly compressing traditional development timelines and reducing reagent waste.

Another key advantage of MiNANO‐form is its simple‐to‐use and contamination‐free operation. Despite being disposable, the system integrates 8 independent channels, enabling the parallel synthesis of 8 distinct formulations per run. This architecture not only prevents cross‐contamination but also significantly boosts throughput, making it ideally suited for high‐throughput screening tasks. Moreover, MiNANO‐form operates with minimal waste/dead volume, nearly all input reagents are used, further contributing to cost‐efficiency. The minimum working volume per formulation is 100–400 µL, dependent on the cartridge, sufficient for comprehensive physicochemical and biological characterization, including particle size, PDI, zeta potential, EE, and cellular assays such as uptake or transfection. To demonstrate the cost‐saving potential, we benchmarked MiNANO‐form against a commercial microfluidic platform, Ignite.^[^ ^41^ ^]^ For a 200‐formulation screen using an mRNA concentration of 0.12 mg mL^−1^, MiNANO‐form consumes ≈9.6 mg of mRNA in 400 µL, costing around €9600 (based on GFP mRNA priced at €1000/mg). Lipid costs add about €200, resulting in a total cost of €9800. In contrast, Ignite requires a minimum of 1 mL per formulation, consuming 24 mg of mRNA and costing €24000 for mRNA and €500 for lipids—a total of €24500. Thus, MiNANO‐form achieves a cost reduction of ≈58%, while retaining high analytical value and reducing material waste. MiNANO‐form processing volume can be further reduced to 100 µL for effective control of synthesis using an aerofoil mixing structure, which makes a cost reduction up to a quarter of it. This efficiency and small volume processing make it particularly advantageous for studies involving expensive or limited‐availability nucleic acids, such as personalized mRNA vaccines and gene therapy candidates.

Looking forward, the high‐throughput platform established here provides a robust foundation for generating large, high‐quality datasets. Such datasets could, in future studies, support the development of machine learning models to further accelerate formulation optimization. While the detailed integration of AI is beyond the scope of the present work, it represents a promising avenue for subsequent research building on this platform.

## 4 Experimental Section

### Materials

SU8 2050 and SU8 Developer were ordered from Kayaku Advanced Materials (Massachusetts, United States). PDMS was purchased from Ellsworth Adhesives (Ireland). DDAB, SM‐102, ALC‐0315, DSPC, and DMG‐PEG2000 were sourced from Avanti Polar Lipids Inc. (Alabama, United States). Cholesterol, ethanol, Tris buffer, and sodium acetate buffer were purchased from Sigma‐Aldrich (Darmstadt, Germany). GFP pDNA, GFP mRMA, and Cy3 labelled siRNA were acquired from GenScript Biotech. Minimum Essential Medium Eagle (MEM) and DMEM 6249 were purchased from Merck Life Science. Fetal bovine serum (FBS), alamarBlue Cell Viability Reagent, NucBlue Live ReadyProbes Reagent, penicillin/streptomycin (p/s), Quant‐it RiboGreen Reagent and RNA Assay Kit, and Quant‐iT PicoGreen dsDNA Assay Kits were ordered from Thermo Fisher Scientific (Waltham, MA). CFBE41o‐human CF bronchial epithelial (CFBE, RRID: CVCL_6901) and A549 Cell Line human (RRID: CVCL_0023) were purchased from Merck. HEK293 cells (RRID: CVCL_0045) were purchased from American Type Culture Collection (Manassas, Virginia, United States).

### Evaluation of Mixing Performance by CFD Simulations and Experimental Tests

PDMS cartridges were used to evaluate the mixing performance of the proposed microchannel design through CFD simulations in COMSOL (Figure S1a, Supporting Information) and experimental tests (Figure S1b, Supporting Information). Flow rates of 180, 720, 1440, and 2880 µL min^−1^ were tested, corresponding to Reynolds numbers (Re) of 12.3, 49.1, 98.3, and 196.6 at the flow junction and outlet of the microfluidic cartridge—remaining within the laminar flow regime (Re < 2000). A fixed flow rate ratio (FRR) of 3:1 was maintained. Mixing experiments used a Rhodamine‐dyed solution, commonly employed as a tracer.^[^ ^49^ ^]^ Mixing performance was quantified using the mixing performance value, calculated from the standard deviation of normalized grayscale values representing concentration.

The standard deviation of concentration in the unmixed state, denoted as *s.d._max_*, was then calculated. In detail, the mixing performance of two miscible liquids was evaluated based to the mass fraction across the section. For ease of calculation, concentration was usually used in place of mass fraction, with the concentrations of the two liquids were set to 0 and 1, respectively. The standard deviation (*s*.*d*.) of the concentration distribution for one liquid across the selected section was calculated using Equation (1):

(1) $$s.d.=\sqrt{\frac{\sum_{i=1}^{n}{\left(C_{i}-\bar{C}\right)}^{2}}{n}}$$

where *C_i_* was the concentration in the *i*th cell, *n* was the number of cells, and $\bar{C}$ ws the average concentration across the section. It is worth noting that the standard deviation in Equation (1) satisfies the condition 0 < *s.d*. < *s.d._max_*. When the concentration of each cell across the section is the same, *s. d*. = 0 indicates complete mixing. Conversely, *s.d. = s. d. _max_* indicates no mixing. The mixing performance can then be quantified and normalized using Equation (2):

(2) $$Mixing\;performance=1-\frac{s.d.}{s.d._{max}}=1-\frac{1}{s.d._{max}}\sqrt{\frac{\sum_{i=1}^{n}{\left(C_{i}-\bar{C}\right)}^{2}}{n}}$$

In this context, *mixing performance* = 0 indicates no mixing, while *mixing performance* = 1 represents complete mixing. The standard deviation was normalized to evaluate mixing efficiency, where a value of 0 corresponds to no mixing and a value of 1 indicates complete mixing. The results provided a visual representation of the mixer's performance.

The Reynolds number (*Re*) is a dimensionless parameter that serves as a key indicator of laminar flow, representing the ratio of inertial forces to viscous forces. It is defined by Equation (3):

(3) $$Re=\frac{\rho UD_{h}}{\mu }=\frac{UD_{h}}{\upsilon }$$

Here, *ρ, U, D_h_, µ*, and *ν* represent the fluid density, average flow velocity, hydrodynamic diameter of the channel, dynamic viscosity, and kinematic viscosity, respectively. The hydrodynamic diameter (*D_h_*) is defined as the ratio of the cross‐sectional area of the channel to its wetted perimeter.

### Fabrication of Plastic Microfluidic Cartridge

**Figure 8:**
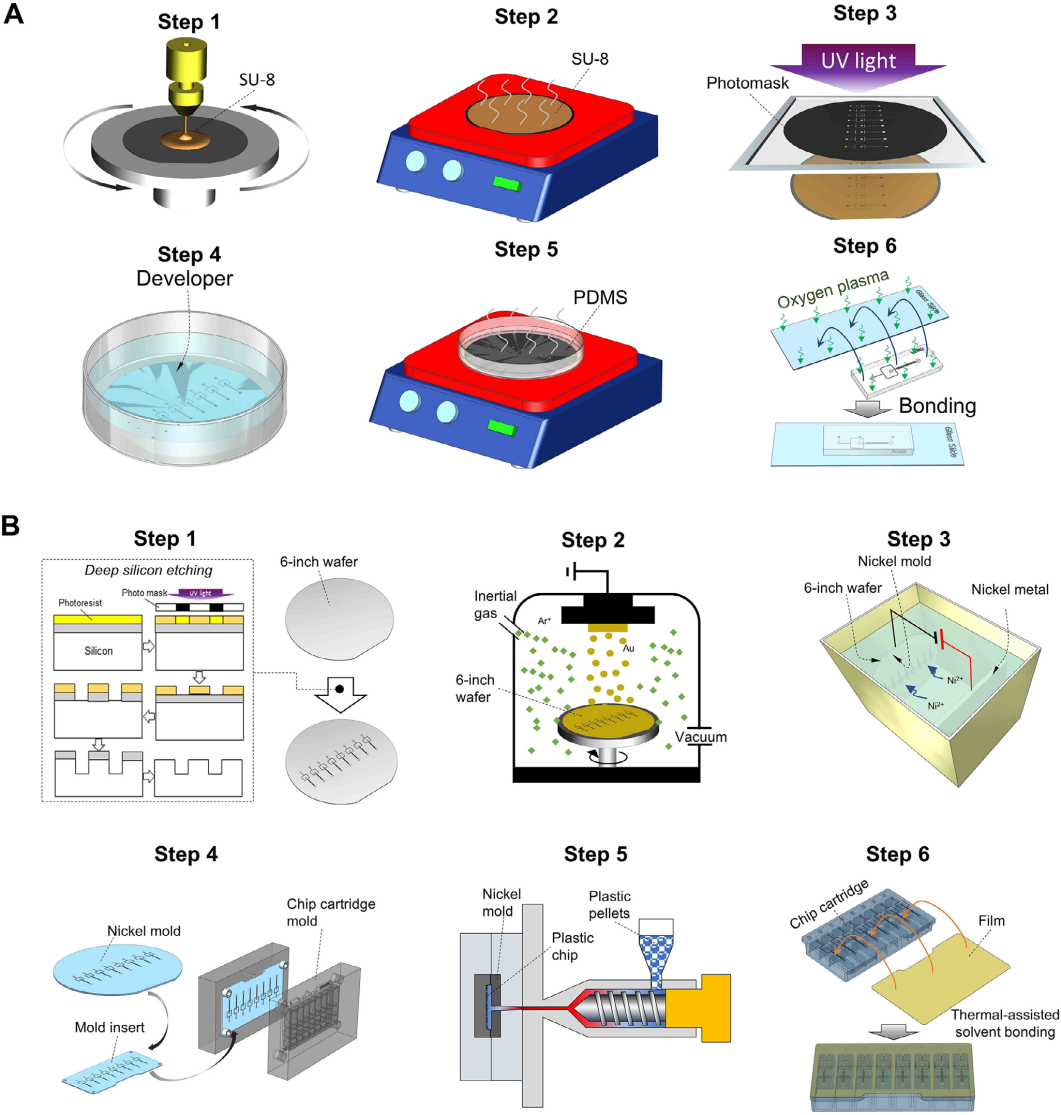
Workflow of PMDS cartridge and plastic cartridge fabrication. A) Workflow of PMDS chip fabrication. Step 1: Spin‐coating of SU‐8 photoresist onto a silicon wafer. Step 2: Soft baking of SU‐8 photoresist to evaporate solvent. Step 3: UV exposure through a photomask to define the chip pattern. Step 4: Development of the exposed SU‐8 to reveal the microchannel structures. Step 5: Casting and curing of PDMS on the patterned wafer. Step 6: Bonding of the cured PDMS chip to a glass slide; B) Workflow for high‐throughput plastic chip fabrication. Step 1: Fabrication of a silicon mask via deep silicon etching. Step 2: Deposition of a conductive layer using physical vapor deposition (PVD). Step 3: Nickel mold fabrication through electroforming. Step 4: Mold insert and chip cartridge fabrication using CNC machining. Step 5: Plastic chip production via injection molding. Step 6: Chip bonding using thermal‐assisted solvent bonding.

The microchannels with aerofoil structures were first prototyped in PDMS cartridges using a six‐step process (**Figure** **8**A). Once the optimal design is identified, plastic cartridges were fabricated using a six‐step process as detailed (Figure 8B). A deep‐etched silicon mask was created on a 6‐inch wafer, followed by PVD deposition of Ti/Au conductive layers. Nickel electroforming replicated the microstructure, and KOH etching yielded a pure nickel mold. To improve performance, Ni‐PTFE nanocomposites were used. The mold was CNC‐processed and assembled with the cartridge frame. COC cartridges were fabricated by micro‐injection molding under the following conditions: nozzle temperature, 270 °C; cylinder zone temperatures, 280 °C (TH1), 275 °C (TH2), 270 °C (TH3), and 260 °C (TH4); mold cavity and core temperatures, 95 °C. Injection pressures were applied in three stages (120, 120, and 110 kg cm^−2^ for Stages I–III, respectively), with injection speeds of 30 mm/s (Stage I) and 40 mm/s (Stage II). The resulting COC cartridges exhibited a surface flatness error of less than µm surface flatness error. Finally, channels were sealed using solvent vapor‐assisted thermal bonding: COC films were exposed for 2 min at a distance of 2 cm above a vapor mixture of 60% cyclohexane and 40% acetone, followed by bonding at 70 °C under a thermal press.^[^ ^50^ ^]^ Cartridges were inspected for clogs, bonding uniformity, and defects.

### Synthesis and Characterization of Empty LNPs

The consistency of the high‐throughput system was evaluated using three different ionizable/cationic lipids: DDAB, SM‐102, and ALC‐0315. The lipid components, including the ionizable/cationic lipid, DSPC, cholesterol (Chol), and DMG‐PEG2000, were dissolved in ethanol at an ionizable/cationic lipid concentration of 0.005 mmol mL^−1^, following a molar ratio of 50:10:38.5:1.5 to form the organic phase. The aqueous phase consisted of either 10 mM Tris buffer (pH 7) or 25 mM sodium acetate buffer (pH 5). Tris buffer was used to prepare DDAB‐LNPs, while sodium acetate buffer was used for SM‐102‐LNPs and ALC‐0315‐LNPs. For LNP synthesis, 300 µL of the aqueous phase was transferred into inlet 1, while 100 µL of the organic phase was transferred into inlet 2 of the microfluidic cartridge. The air pressure was adjusted according to the specific aqueous phase solution, maintaining a flow rate ratio of 3:1 and a total flow rate of 2 mL min^−1^. The collected LNPs were diluted fivefold with PBS and characterized using the Litesizer DLS 500 (Anton Paar, Austria) to determine size and PDI.

By applying the above‐mentioned procedure at various total flow rates, 0.5, 1, 2, and 4 mL min^−1^, the effect of total flow rate on LNP formation was investigated using MiNANO‐form. Their size and PDI were also measured as explained above, MiNANO‐form.

To assess the scalability and performance consistency of the MiNANO‐scale system, LNPs were synthesized using the SM‐102 lipid formulation under different flow rates. The organic phase used the same solution from the previous step. The system was tested at total flow rates of 5, 10, 20, and 50 mL min^−1^, with a constant aqueous‐to‐organic flow rate ratio of 3:1. Each solution was delivered to the scaled‐up microfluidic cartridge via peristaltic pumps. Upon mixing, the LNPs were collected and diluted fivefold in PBS. Size and PDI of the collected LNPs were measured using the Litesizer DLS 500.

### Nucleic Acid‐Loaded LNPs Synthesis

To evaluate the performance of LNPs in nucleic acid delivery, mRNA, pDNA, and siRNA were encapsulated, and their transfection and uptake efficiencies were tested using HEK, A549, and CFBE cells. GFP mRNA was dissolved in Tris buffer (10 mM, pH 7) or sodium acetate buffer (25 mM, pH 5) at a concentration of 0.16 mg mL^−1^. GFP pDNA was similarly prepared at a concentration of 0.08 mg mL^−1^, while Cy3‐labeled siRNA was prepared at 2 nmol mL^−1^. LNPs were synthesized using the same lipid formulations as described previously. For nucleic acid encapsulation, 300 µL of the aqueous phase containing the nucleic acid was introduced into inlet 1, while 100 µL of the organic phase was introduced into inlet 2 of the microfluidic cartridge. The flow rate ratio was maintained at 3:1, with a total flow rate of 2 mL min^−1^. The collected LNPs were diluted fivefold with PBS and characterized using the Litesizer DLS 500. Encapsulation efficiency (EE) of mRNA‐LNPs and pDNA‐LNPs was determined using the Quant‐iT RiboGreen RNA Assay Kit (for mRNA‐LNPs), Quant‐iT PicoGreen dsDNA Assay Kits (for pDNA‐LNPs) and calculated using the following equation:^[^ ^51^ ^]^

(4) $$\begin{array}{ccc} & & EE=\\ & & \frac{Total\;mRNA/pDNA\;concentration-uncapsulated\;mRNA/pDNA}{Total\;mRNA/pDNA\;concentration}\times 100\% \end{array}$$

### Cell Culture

HEK and A549 cells were cultured in DMEM supplemented with 10% FBS and 1% penicillin/streptomycin. CFBE41o‐ cells were maintained in MEM supplemented with 10% FBS, 1% penicillin/streptomycin, 1% MEM non‐essential amino acids, and 1% glutamine. Cells were seeded at densities of 62500 cells/cm^2^ for HEK cells and 31250 cells/cm^2^ for A549 and CFBE41o‐ cells in 96‐well plates. Cells were incubated at 37 °C with 5% CO_2_ before transfection and uptake experiments.

### Cell Transfection

LNPs containing mRNA or pDNA were purified via two rounds of ultrafiltration. Culture medium was replaced with a mixture containing 25 µL of mRNA‐LNPs or pDNA‐LNPs and 175 µL of fresh culture medium. Each condition was tested in triplicate wells. GFP expression was assessed 48 hours post‐transfection using an Olympus IX81 fluorescence microscope. Fluorescent images were analyzed using ImageJ Fiji software for semi‐quantitative analysis of fluorescence intensity.

### Cell Viability

Cell viability was assessed 48 hours post‐transfection. The culture medium containing mRNA‐LNPs or pDNA‐LNPs was removed, and cells were washed with HBSS. A 100 µL solution of 10% alamarBlue in HBSS was added to each well and incubated for 1.5 hours. Absorbance was recorded at 570 and 600 nm using a SpectraMax M3 multi‐plate reader (Molecular Devices, San Jose, CA). Untreated cells served as the normalization control (100% viability). Background fluorescence was subtracted from wells containing only alamarBlue reagent, and cell viability was calculated using the following equation:

(5) $$\frac{Absorbance\;of\;treated\;cells}{Absorbance\;of\;untreated\;cells}\times 100\%$$

### Cellular Uptake

To assess cellular uptake, culture medium was replaced with a mixture of 25 µL siRNA‐LNPs and 175 µL fresh culture medium. Each condition was tested in triplicate wells. Following incubation, cells were washed with PBS and stained with NucBlue Live ReadyProbes Reagent. After 20 min, cells were washed with PBS, and fluorescence images were captured using an Olympus IX81 fluorescence microscope

### Statistical Analysis

All experiments were performed in triplicate unless otherwise specified. Data were presented as mean ± standard deviation (±SD). One‐way ANOVA with Dunnett's multiple‐comparison tests was used to analyze the difference for different groups using Prism 8.0 (GraphPad). A difference was considered significant if *p* < 0.05 (^*^ *p* < 0.05, ^**^ *p* < 0.01, ^***^ *p* < 0.001, ^****^ *p* < 0.0001).

## Conflict of Interest

The authors have filed a patent application (Application No. GB2303464.8) related to this work.

## Author Contributions

D.L., M.Y. contributed equally to this work. N.Z. conceived the project, designed the cartridge and entire platform, and secured funding. D.L. and M.Y. designed and optimized the aerofoil structures and the MiNANO‐form platform, and evaluated LNP performance using MiNANO‐form. Y.Z. developed the MiNANO‐scale system, fabricated the corresponding cartridge, and tested LNPs using MiNANO‐scale. A.M. fabricated the plastic cartridge. T.G. prepared the mold insert. M.Y., L.Y., and X.W. conducted the cell‐based experiments. W.W. contributed to the conceptual development of the project. N.Z., D.L., M.Y., and A.M. wrote the manuscript. All authors contributed to the discussion and interpretation of the results.

## Supporting information



Supporting Information

## Data Availability

The data that support the findings of this study are available in the supplementary material of this article.
