# Supplementary material for: Unique Aerofoil‐Structured Microfluidics for High Throughput Lipid Nanoparticle Formulation Screening and Scale‐up
Source: Adv Sci (Weinh). 2025 Oct 28;13(6):e11222. doi: 10.1002/advs.202511222 (PMC12866714; doi:10.1002/advs.202511222)
Supplement: Supplementary file 1 — Supporting Information [file ADVS-13-e11222-s001.docx]

Supporting Information for

Unique Aerofoiled Microfluidics for High Throughput Lipid Nanoparticle Formulation Screening and Scale-up

Dongsheng Liu^1, 2, 3,^ †, Mingzhi Yu^1,^ †, Yuguo Zhang^1^, Allen Mathew^1^, Tianyu Guan^1^, Liang Yao^4^, Xianqing Wang^4^, Wenxin Wang^4^, Nan Zhang^1*^

^1^Centre of Micro/Nano Manufacturing Technology (MNMT-Dublin), School of Mechanical & Materials Engineering, University College Dublin, D04 V1W8, Ireland

^2^Department of Aerospace and Mechanical Engineering, South East Technological University, Carlow R93 V960, Ireland.

^3^The Centre for Research and Enterprise in Engineering (engCORE), South East Technological University, Carlow R93 V960, Ireland

^4^Charles Institute of Dermatology, School of Medicine, University College Dublin, D04 V1W8, Dublin, Ireland

†These authors contributed equally: Dongsheng Liu, Mingzhi Yu.

*Corresponding author. E-mail: nan.zhang@ucd.ie


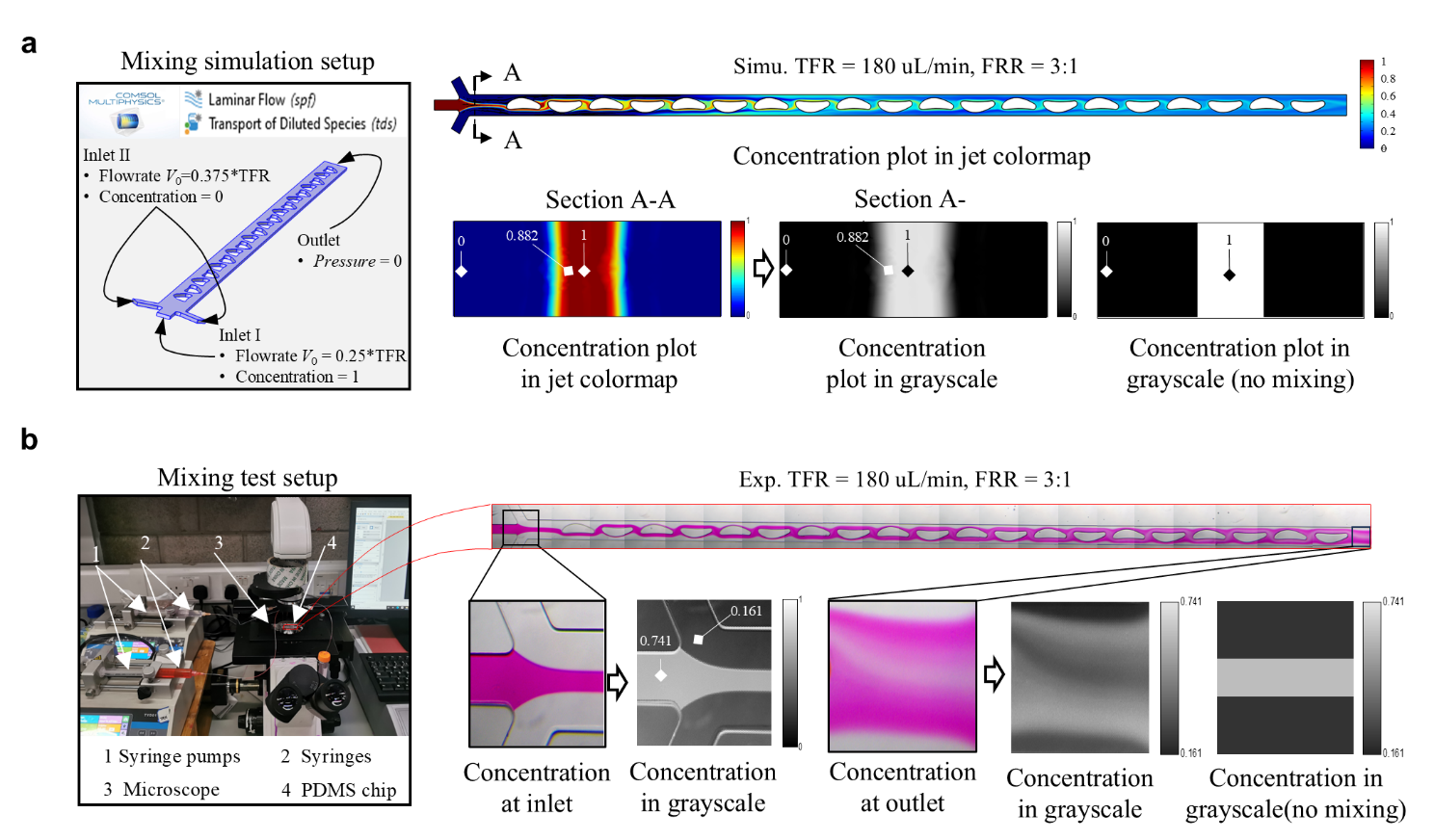


Fig. S1. Mixing evaluation by CFD simulation and experimental testing. (a) CFD-based mixing simulation with corresponding image processing and calculation of mixing performance (MP); (b) Experimental mixing test with image processing and calculation of mixing performance (MP).

| (a)  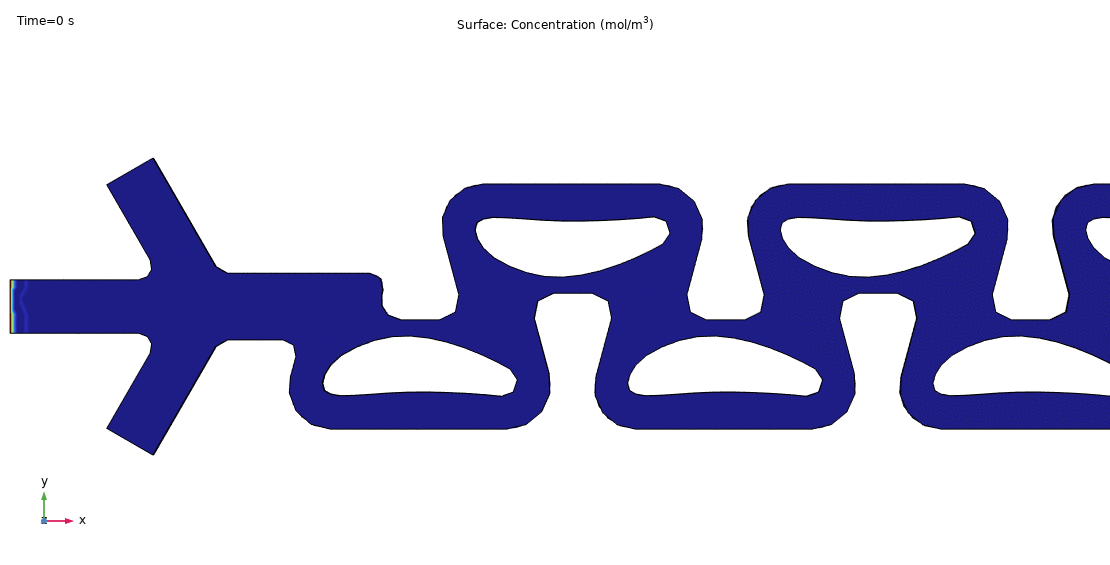 | (b)  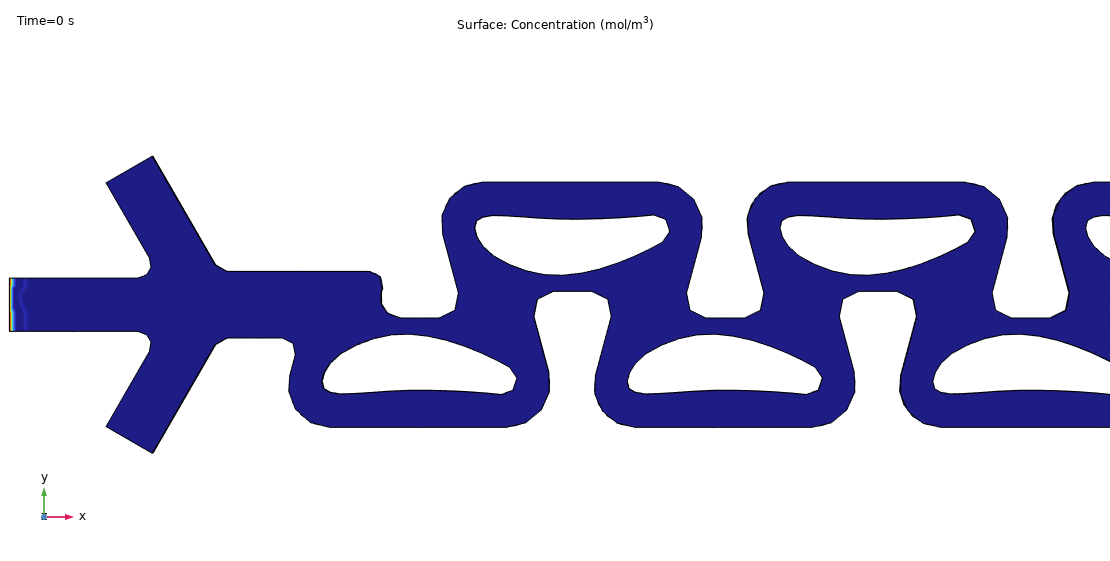 |
| --- | --- |
| (c)  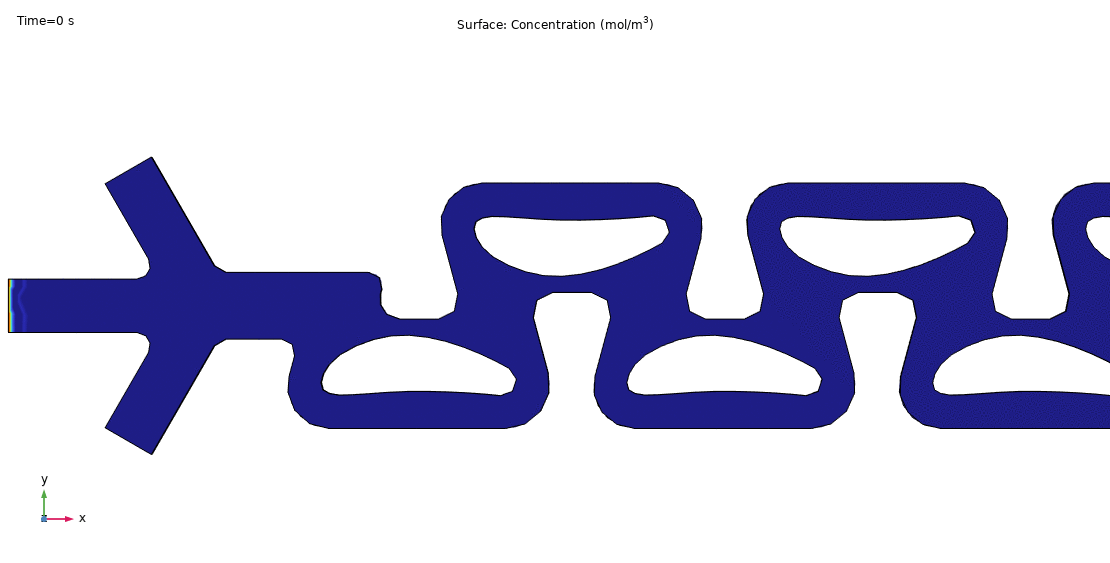 | (d)  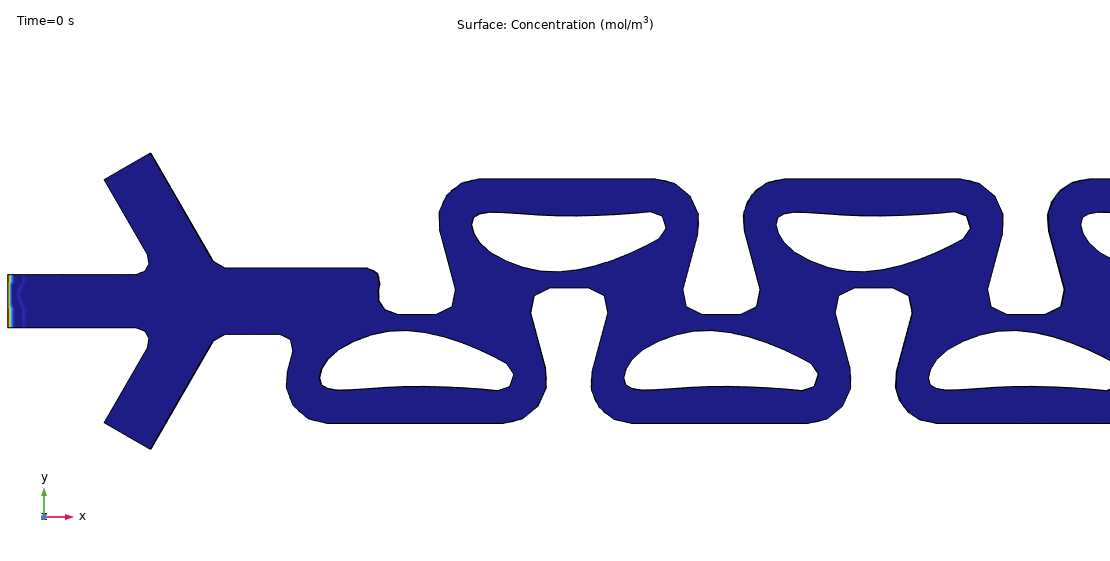 |

Fig. S2. Transient CFD simulations at different total flow rates (TFR). (a) 180 mL/min, stable state reached at 0.088 s; (b) 720 mL/min, stable state reached at 0.019 s; (c) 1440 mL/min, stable state reached at 0.0125 s; (d) 2880 mL/min, stable state reached at 0.0089 s.

**
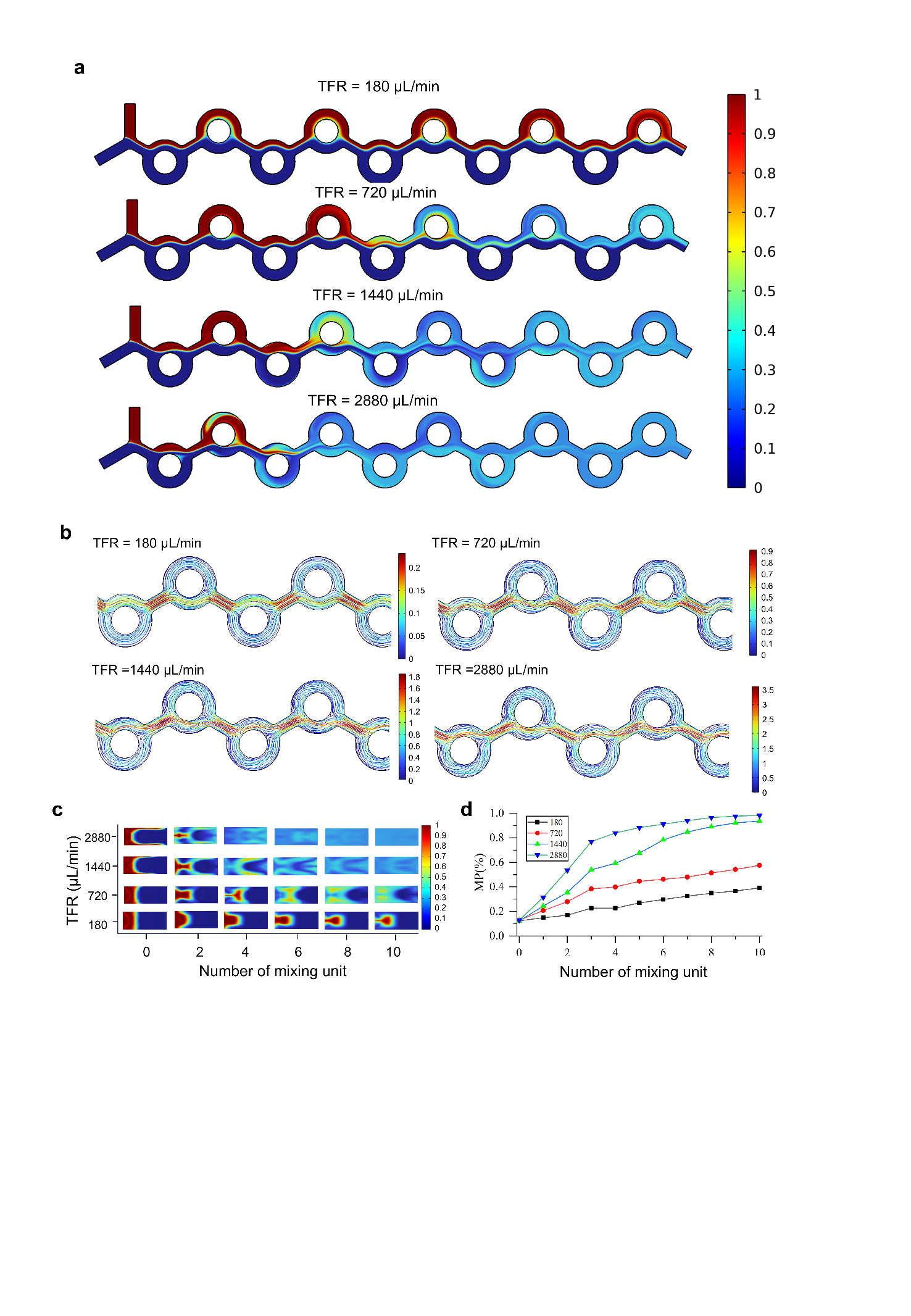
**

Fig. S3. Simulation results for Dean vortex bifurcating mixer (DVBM) at various TFRs. (a) Concentration results; (b) Velocity streamline plots; (c) Cross section of concentration at outlet; (d) Mixing performance (MP) of cross section at outlet. Note: The length of one DVBM mixing unit is 1.1612 mm. For the optimal chip configuration (105/60/120/100), the mixing unit length is 1.1463 mm.


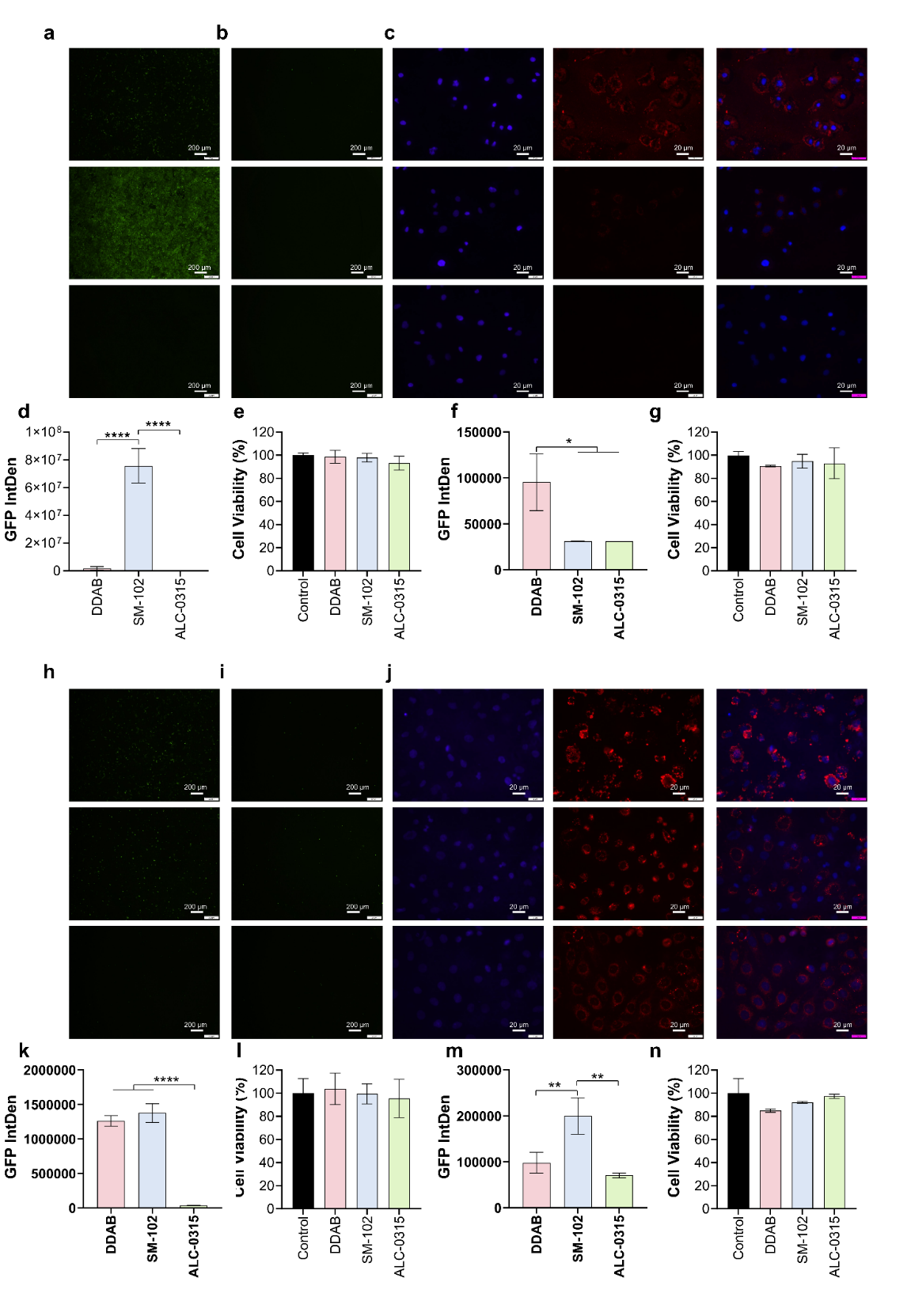


Fig. S4. Cellular evaluation using A549 and CFBE cells. (a) Representative fluorescence microscopy images of GFP expression in A549 cells transfected with GFP mRNA; (b) Representative images of GFP expression in A549 cells transfected with GFP pDNA; (c) Uptake of Cy3-labeled siRNA by A549 cells; (d) Quantification of GFP fluorescence intensity in A549 cells (GFP mRNA); (e) Cell viability after GFP mRNA transfection in A549 cells; (f) Quantification of GFP fluorescence intensity in A549 cells (GFP pDNA); (g) Cell viability after GFP pDNA transfection in A549 cells; (h) Representative fluorescence images of GFP expression in CFBE cells transfected with GFP mRNA; (i) Representative images of GFP expression in CFBE cells transfected with GFP pDNA; (j) Uptake of Cy3-labeled siRNA by CFBE cells; (k) Quantification of GFP fluorescence intensity in CFBE cells (GFP mRNA); (l) Cell viability after GFP mRNA transfection in CFBE cells; (m) Quantification of GFP fluorescence intensity in CFBE cells (GFP pDNA); (n) Cell viability after GFP pDNA transfection in CFBE cells.


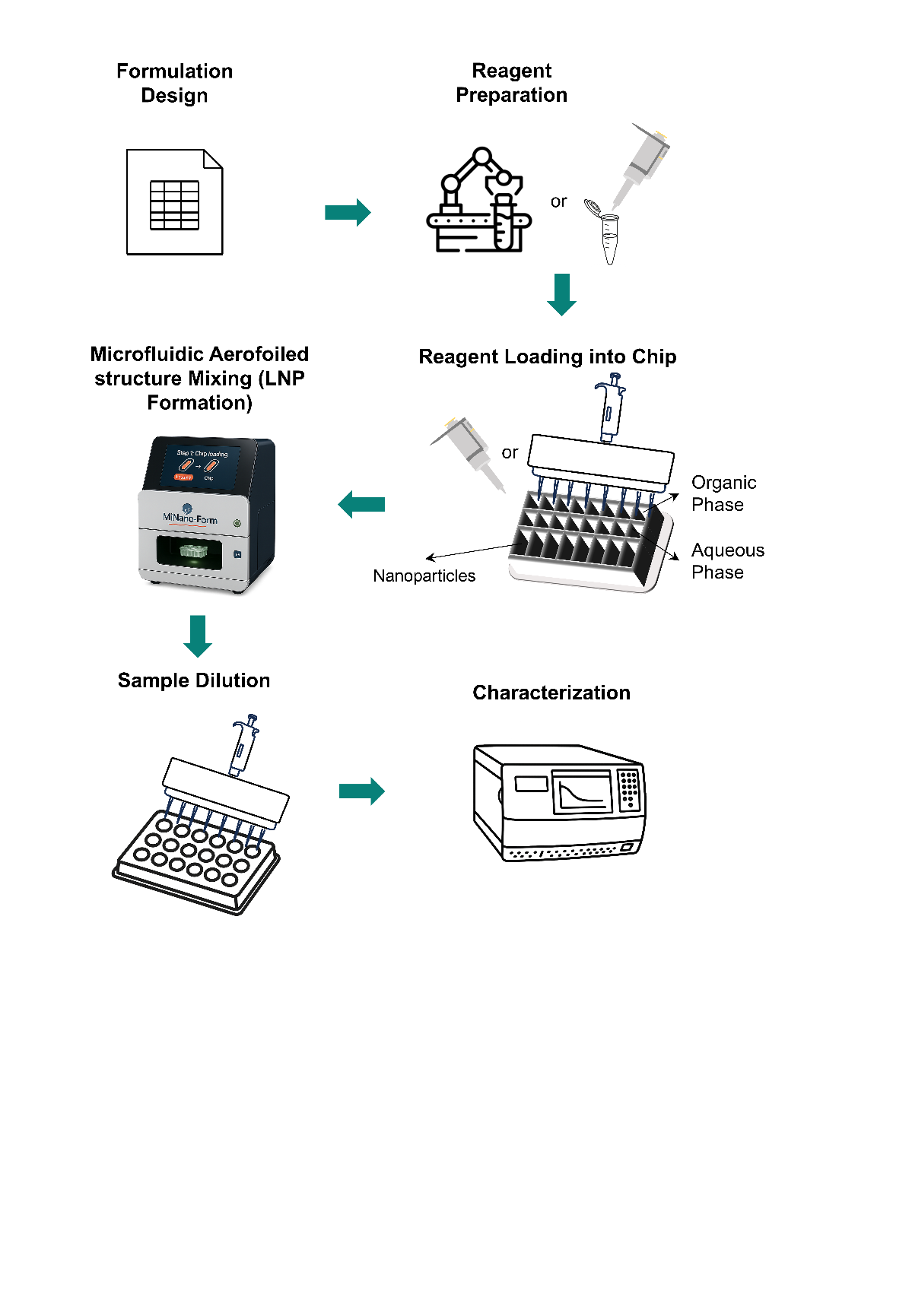


Fig. S5. Workflow of high-throughput LNPs synthesis using a microfluidic chip system

Table S1. Consistency test results

| DDAB-LNP results | | |
| --- | --- | --- |
| Channel | Average size | Average PDI |
| A | 86.82 | 0.093 |
| B | 83.84 | 0.084 |
| C | 87.39 | 0.049 |
| D | 87.6 | 0.098 |
| E | 87.22 | 0.094 |
| F | 87.53 | 0.048 |
| G | 86.17 | 0.064 |
| H | 91.68 | 0.156 |
| Mean value | 87.28 | 0.086 |
| Standard deviation | 2.17 | 0.0348 |
| Coefficient Of Variation (%) | 2.48 | 40.54 |
| SM-102-LNP results | | |
| Channel | Average size | Average PDI |
| A | 38.96 | 0.161 |
| B | 38.73 | 0.151 |
| C | 40.63 | 0.165 |
| D | 43.22 | 0.196 |
| E | 43.66 | 0.209 |
| F | 42.26 | 0.17 |
| G | 41.55 | 0.161 |
| H | 41.19 | 0.126 |
| Mean value | 41.275 | 0.234 |
| Standard deviation | 1.80 | 0.0257 |
| Coefficient Of Variation (%) | 4.37 | 15.36 |
| ALC-0315-LNP results | | |
| Channel | Average size | Average PDI |
| A | 46.11 | 0.085 |
| B | 46.66 | 0.135 |
| C | 50.97 | 0.122 |
| D | 50.11 | 0.14 |
| E | 50.07 | 0.138 |
| F | 51.85 | 0.151 |
| G | 47.45 | 0.163 |
| H | 51.31 | 0.106 |
| Mean value | 49.32 | 0.130 |
| Standard deviation | 2.24 | 0.0250 |
| Coefficient Of Variation (%) | 4.54 | 19.25 |
